# rAvis: An R-Package for Downloading Information Stored in Proyecto AVIS, a Citizen Science Bird Project

**DOI:** 10.1371/journal.pone.0091650

**Published:** 2014-03-13

**Authors:** Sara Varela, Javier González-Hernández, Eduardo Casabella, Rafael Barrientos

**Affiliations:** 1 Department of Ecology, Faculty of Science, Charles University, Prague, Czech Republic; 2 Departamento de Ecologia, Instituto de Ciencias Biologicas, Universidade Federal de Goiás, CxP 131, Goiania, Goiás, Brasil; 3 Toledo, Spain; 4 ACCONTE Group, Actividades de Consultoría y Telecomunicaciones, Tres Cantos, Spain; 5 Departamento de Ciencias Ambientales, Facultad de Medio Ambiente, Universidad de Castilla-La Mancha, Toledo, Spain; UCLA, United States of America

## Abstract

Citizen science projects store an enormous amount of information about species distribution, diversity and characteristics. Researchers are now beginning to make use of this rich collection of data. However, access to these databases is not always straightforward. Apart from the largest and international projects, citizen science repositories often lack specific Application Programming Interfaces (APIs) to connect them to the scientific environments. Thus, it is necessary to develop simple routines to allow researchers to take advantage of the information collected by smaller citizen science projects, for instance, programming specific packages to connect them to popular scientific environments (like R). Here, we present rAvis, an R-package to connect R-users with Proyecto AVIS (http://proyectoavis.com), a Spanish citizen science project with more than 82,000 bird observation records. We develop several functions to explore the database, to plot the geographic distribution of the species occurrences, and to generate personal queries to the database about species occurrences (number of individuals, distribution, etc.) and birdwatcher observations (number of species recorded by each collaborator, UTMs visited, etc.). This new R-package will allow scientists to access this database and to exploit the information generated by Spanish birdwatchers over the last 40 years.

## Introduction

During the past several decades, developers have focused their attention on constructing web repositories to store and share biological information. On the one hand, there are online repositories with information generated by scientists, like specimens collected for museums and herbariums, fossil records or genetic data (e.g. GBIF: http://gbif.org, NOW: http://helsinki.fi/science/now/, GeneBank: http://ncbi.nlm.nih.gov/genbank/). On the other hand, there are web sites that store biological information collected by non-scientists, or so-called ‘citizen science’.

**Figure 1:**
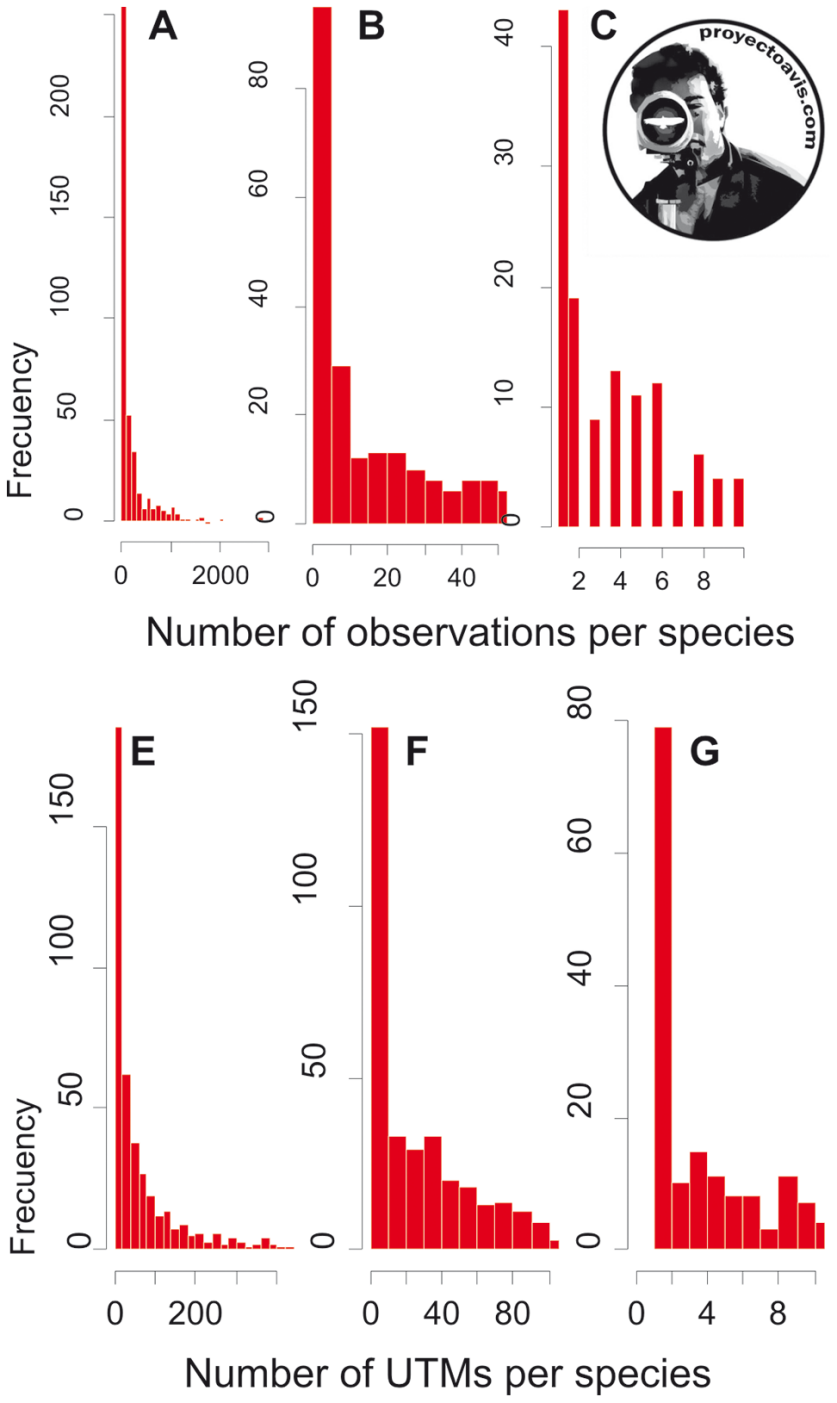
Data collected by amateur birdwatchers and stored in Proyecto AVIS show the same patterns as data collected by scientists, like scale invariant relationship of the frequency distribution of the number of observations per species (A-B-C) and scale invariant relationship of the frequency distribution of the number of UTMs per species (E-F-G).

Citizen science has proven to be an appropriate method to provide researchers with valuable information [1]–[3], and is increasingly used as an adequate way to sample species occurrences and distributions [4], to collect data to investigate urban ecology [3], [5], [6], or to collect data on bird biology, ecology and diversity [7]–[9]. In our case, data stored in Proyecto AVIS, our citizen science project to collect data from amateur Spanish ornithologists, show the same general patterns described by scientists based on their own samples and field experiments. Power law distributions of species/area [10] and species/abundance [11] have been detected (Figure 1), suggesting that the data stored in Proyecto AVIS have similar properties to the data collected by scientists.

One of the main characteristics of the citizen science databases is that they are huge. For instance, birdwatchers’ observations stored in the eBird database reached 100,000,000 observations and over 10,000 species (http://ebird.org). As a result, there are terabytes of information about species occurrences (latitude, longitude, altitude, time, habitat, diet, alleles, etc.) stored in online databases that follow different formats and standards of data storage [12], and the challenge now is developing easy strategies to use this information for research [13].

Currently, there are ongoing projects to generate tools to standardize the information stored in those databases (e.g. http://ecodataretriever.org) and to develop R-packages to connect online biological databases to the R-environment (http://ropensci.org/). As a consequence, large international databases are now being made available through R using packages like *rebird* [14], *rfishbase* [15], *rgbif* [16] or *rvertnet* [17] (connecting R with eBird, Fishbase, GBIF and VertNet databases, respectively). All of these new data exponentially increase our capabilities to answer questions about species conservation, global change, macroecology and biogeography.

R is an open source and collaborative framework (http://.r-project.org/), and is one of the most used environments for analyzing biological data and for developing scientific software [18]. Many young scientists are becoming advanced R-users (but see [19]). Thus, R is becoming a standard environment for developing easy-to-use (and re-use) functions and for sharing them with the academic community. For all of these reasons, we decided to build an R package to directly download the information stored in Proyecto AVIS from the R environment, in order to promote the use of the data stored in this database within the growing scientific R-community.

### Proyecto AVIS

Each citizen science project stores singular and, consequently, important information [6], [9], [20]–[22]. Proyecto AVIS (http://proyectoavis.com) is a citizen science project born in August 2005 with the idea of collecting the data stored in the field notebooks of amateur Spanish ornithologists and sharing them with both other amateur ornithologists and the scientific community. More than one hundred collaborators, including several NGOs, have been actively participating in the project uploading their bird observations. Overall, the database contains records over 40 years (1973–2013), stores 82,503 records, totalling 4,739,171 individuals from 413 species, which represents 90% of the total number of species recorded in Spain. In addition, it contains information from 1,717 different UTMs (squares of 10×10 km), representing 30% of the Spanish territory (query to the database: November 2013).

The Proyecto AVIS database and web page were built using open source software (MySQL, Perl, Apache) and free GIS layers. Proyecto AVIS requires five mandatory fields for each bird observation: ‘species’, ‘number of individuals’, ‘observation period’, ‘date’ and ‘UTM 10×10 km square’, plus several optional fields that include variables like ‘hour’, ‘sex’, ‘age’ or ‘habitat’. To standardize the taxonomy, the bird species list follows the Bird List of Spain from SEO/BirdLife [23]. Bird occurrences in the Proyecto AVIS database are georeferenced using the projected UTM 10×10 km square system and the MGRS labelling convention (Military Grid Reference System). The UTM/MGRS is the standard system for mapping species occurrences in Spain and is the system used by the Spanish bird atlases [24], [25]. To help users identify the UTMs in which they recorded the species, the web application includes an easy-to-use tool to geo-referenced the observations based on a Google Maps ™ routine.

The Proyecto AVIS web page (http://proyectoavis.com) includes several user-friendly tools for exploring the database, like summaries of the bird observations or graphics of the species records throughout the year, and it allows registered users to download detailed information about the species observations to Excel files. However, although the database is already available on the Internet, its use for research has not been properly exploited. Proyecto AVIS lacks a specific package to connect the web repository with the R-environment, and we believe that this fact has prevented scientists from using Proyecto AVIS information.

### Description of the package

rAvis exclusively contains R code, which maximizes its portability across platforms, and it works in Unix-like and Windows operating systems. The rAvis functions have been optimized following the standards criteria for software quality [26], [27] and they are accessible through GitHub (https://github.com/javigzz/rAvis). Bugs can be reported using GitHub; https://github.com/javigzz/rAvis/issues. rAvis is freely available on the Comprehensive R Archive Network; CRAN (http://cran.r-project.org/) and complete information about rAvis, its functions and their parameters is available in the package help.

rAvis uses functions from other R-packages to get and plot the data stored in Proyecto AVIS. Namely, R-libraries stringr [28], XML [29], tools [30], RCurl [31], scrapeR [32] and gdata [33] are used to download the bird observations; maptools [34], raster [35] and rgdal [36] to plot the GIS files; and, finally, scales [37] is used to plot bird occurrences with a transparency.

#### Exploring Proyecto AVIS

**Figure 2:**
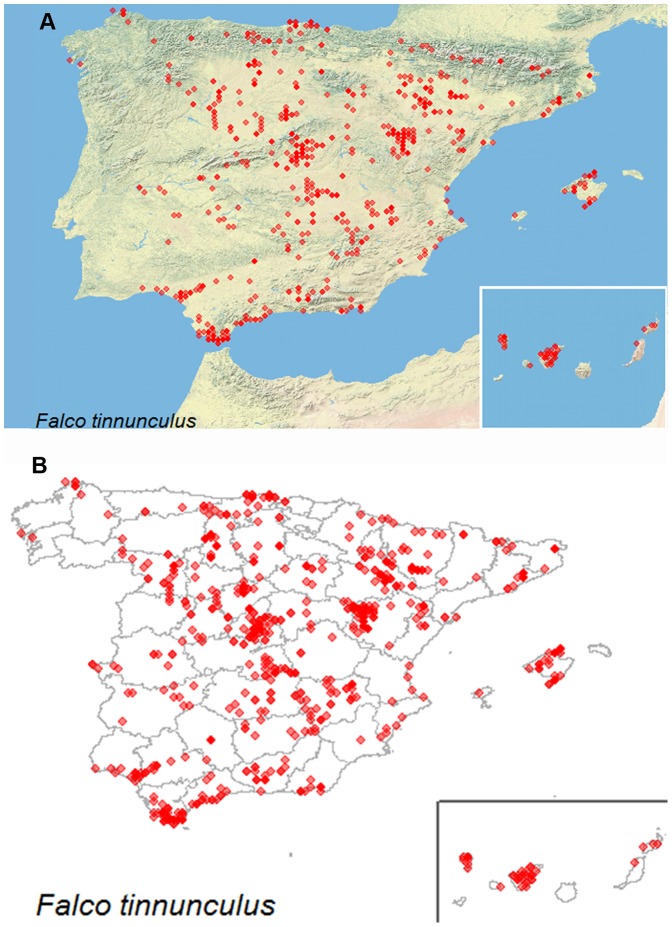
Outputs of the function *avisMapSpecies* setting the parameter map as ‘phys’ (A), or ‘admin’ (B) with the *Falco tinnunculus* records as an example.

We developed several functions to explore the database in an easy and visual way and other functions to download the selected information (see Table 1 and run the example). First, *avisSpeciesSummary* allows users to download a table with a summary of the records stored in Proyecto AVIS aggregated by species: number of observations of each species, number of individuals recorded, number of different UTMs (10×10 km) with observations, number of birdwatchers that recorded the species. Second, *avisContributorsSummary* returns a table with a general summary of the records stored in the database aggregated by birdwatcher: number of observations per birdwatcher, number of species observed, number of provinces with data, number of UTMs visited, number of periods of observations. Finally, *avisHasSpecies* checks if a species name exists in Proyecto AVIS and then, *avisMapSpecies* allows users to explore the distribution of the observations of the species by setting the name of the species and selecting the type of map; administrative boundaries ('admin') or physical map ('phys') (Figure 2).

**Table 1:** Descriptions of the functions of the rAvis R-package.

Functions in rAvis	Description
avisHasSpecies	checks if a species name exists in Proyecto AVIS.
avisSpeciesId	Returns the id of the selected species
avisAllSpecies	Returns a list with the species names and the ids of the species in Proyecto AVIS
avisMap	Renders a map for the observations downloaded using avisQuerySpecies
avisMapSpecies	Renders a map for each of the selected species (could be a list of species)
avisSpeciesSummary	Downloads a table with a summary of the records stored in Proyecto AVIS aggregated by species; number of observations of each species, number of individuals recorded, number of different UTMs (10×10 km) with observations and number of birdwatchers that recorded the species
avisQuery	Downloads information using several filters, like Order, Family, Species, Age, Sex, Habitat, etc.
avisQuerySpecies	Wrapper for avisQuery that allows to perform a query for more than one species at once.
avisContributorsSummary	Returns a table with the observations aggregated by birdwatcher.
avisContributorAggregatedObservations	Downloads the information about the observations of a birdwatcher
avisQueryContributor	Wrapper for avisQuery that allows to perform a query for more than one contributor at once.
avisSetup	Allows the user to turn off the information messages provided by the functions using “verbose = FALSE”

For constructing the plots we used free GIS layers. We downloaded the Spanish administrative map from http://.diva-gis.org/, the Spanish UTM map from the Spanish government online map repository http://bscw.rediris.es/pub/bscw.cgi/524254?client_size=1366×580, and the Spanish physical map from http://.openstreetmap.org/ using the R- library OpenStreetMap [38].

#### Advanced queries to Proyecto AVIS

We constructed two main functions to set flexible queries about the species occurrences and the birdwatcher observations: *avisQuerySpecies* and *avisQueryContributor*, respectively. These functions download the information stored in Proyecto AVIS, and are intended to be tuned by the users in relation to their specific objectives. Also, we programmed avisQuery as a flexible function to pass any argument allowed in Proyecto AVIS database. We decided not to predefine queries or to pre-process the data because this would narrow the possibilities for research [12]. Instead, we allow the users to set their own queries to Proyecto AVIS. Arguments include taxonomic levels, like species, family, order; individual characteristics, like age, sex, breeding status; temporal filters, like year and month; or environmental filters, like habitat. Moreover, we added a UTM-latlong conversion to all queries. Thus, the position of the observations is given in two different formats: projected UTMs 10×10 km and geographic coordinates WGS84 (common latitude-longitude coordinates, which are not available in the current web application from Proyecto AVIS). We did not program more specific graphics or statistical analyses because we understand that the purpose of this package is to obtain the biological information stored in Proyecto AVIS and not to re-program statistical algorithms that are already available in other R-packages. We assume that R-users would employ different R-packages for calculating their own statistics and constructing their own plots (see the example).

#### Example

rAvis could be upgraded in future releases. To download the exact version of rAvis that we used in this example run the function install_github from devtools package as follows: install_github("javigzz/rAvis", ref = "v0.1")

Install rAvis from the CRAN and load the package > install.packages ("rAvis") > library(rAvis) > avisSetup (verbose = FALSE) Check if the target species has records in Proyecto AVIS > avisHasSpecies ("Pica pica") Plot the occurrences of the species to explore the data > avisMapSpecies ("Pica pica", maptype = "phys") Download the occurrrences of the species > Pica_pica<- avisQuerySpecies ("Pica pica") Filter the data using avisQuery. For instance, select only records from forests habitats setting habitat = "bosque" (the database is in Spanish) > Pica_pica_forest<- avisQuery (species = "Pica pica", > habitat = "bosque")

Plot the results using avisMAp > avisMap (Pica_pica_forest, label = "Pica pica; Forest") If interested in several species, explore the database using avisMApSpecies > avisMapSpecies (list("Tyto alba", "Athene noctua", > "Bubo bubo", "Strix aluco"), maptype = "phys") Save the maps individually using the tiff function > directory<- "C:/your_directory" > species<- list("Tyto alba", "Athene noctua", > "Bubo bubo", "Strix aluco") > for (x in species){ > tiff (file.path (directory, paste ("/", x, ".tiff", sep = ""))) > avisMapSpecies (x) > dev.off() > }

## Conclusions

We have programmed rAvis, an R-package designed to help researchers explore and download the information stored in Proyecto AVIS. Thus, biogeographers, macroecologists and ornithologists working in spatial ecology or temporal series, in addition to researchers working on citizen science can easily take advantage of the unique data stored in this database for their own research.
